# Buruli Ulcer, Central African Republic

**DOI:** 10.3201/eid1604.090195

**Published:** 2010-04

**Authors:** Fanny Minime-Lingoupou, Narcisse Beyam, Germain Zandanga, Alexandre Manirakiza, Alain N’Domackrah, Siméon Njuimo, Sara Eyangoh, Jane Cottin, Laurent Marsollier, Estelle Marion, Francoise Portaels, Alain Le Faou, Raymond Bercion

**Affiliations:** Pasteur Institute, Bangui, Central African Republic (F. Minime-Lingoupou, N. Beyam, G. Zandanga, A. Manirakiza, A. N’Domackrah, S. Njuimo, A. Le Faou, R. Bercion); Pasteur Center, Yaounde, Cameroon (S. Eyangoh); Central Hospitalier universitaire, Angers, France (J. Cottin, E. Marion); Université d’Angers, Angers (J. Cottin, L. Marsollier, E. Marion); Institute for Tropical Medicine, Anvers, Belgium (F. Portaels)

**Keywords:** Buruli ulcer, Mycobacterium ulcerans, bacteria, letter

**To the Editor:** Buruli ulcer, the third most common mycobacterial disease of humans after tuberculosis and leprosy, is an important disfiguring and disabling cutaneous infection disease caused by *Mycobacterium ulcerans*. Buruli ulcer was declared an emerging skin disease of public health concern by the World Health Organization (WHO) in 1998. Although the disease is known to be associated with swampy areas and environmental changes, the mode of transmission is not yet clearly understood. A possible role for water bugs in the transmission has been postulated in the last 10 years. In this direction, several researchers have proposed that biting water bugs could be vectors for *M. ulcerans* (*1*). *M. ulcerans* produces a potent toxin known as mycolactone (*2*), which lyses dermal cells, leading to the development of continuously expanding ulcers with undermined edges. Surgery is the only treatment for late lesions, which involves excision of necrotic tissues, followed by skin grafting. After such treatment, patients suffer from functional limitations, social stigmatization, and the loss of livelihood (*3*). Antimicrobial drug treatment is available (a combination of rifampin and streptomycin), but it is effective only for early lesions (*4*).

The disease is endemic in rural wetlands of tropical countries of Africa, the Americas, and Asia. Over the past decade, the prevalence of Buruli ulcer was highest in western Africa (*3*,*5*), with an alarming increase in detected cases. In central Africa, foci of Buruli ulcer have been reported in Gabon, Equatorial Guinea, Cameroon, Congo, the Democratic Republic of Congo, and Sudan (*6*), which are all neighboring countries of the Central African Republic (CAR). Surprisingly, in CAR, no cases of Buruli ulcer have been reported so far, even though its presence in this country was suspected in 2006, although not confirmed. This situation motivated us to begin a passive survey in the hospitals of Bangui, the capital of CAR. We report here 2 confirmed cases of Buruli ulcer that were found through this survey. The 2 patients were admitted in April 2007 to Hôpital de l’Amitié, Bangui, CAR, with extensive skin ulcers, which might correspond to Buruli ulcer according to WHO guidelines (*7*). Both patients were farmers from the Ombella M’poko region. They lived on the border of the M’poko River and carried out daily activities in an aquatic environment.

The first patient was a 62-year-old man who had a large ulceration of the right limb (Figure, panel A). Differential diagnosis eliminated other ulcerative diseases such as drepanocytosis, and the patient was HIV negative. For bacteriologic diagnosis, 4 samples were taken with sterile cotton swabs from beneath the undermined edges of the ulcer. *Proteus mirabilis* was isolated from the lesion, and a few acid-fast bacilli were shown by Ziehl-Neelsen (ZN) staining. Unfortunately, 1 week later, the patient died of an unknown cause.

**Figure:**
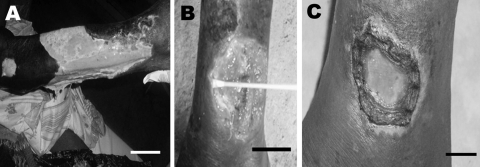
Patient 1: extensive ulcer of the right limb (A). Patient 2: ulcer of the left ankle before treatment (B) and 8 weeks after specific antimicrobial drug therapy (C). Scale bars = 12 cm (A), 5 cm (B), and 2 cm (C).

The second patient was a man of the same age who had an ulceration 6.5 cm in diameter on the left ankle (Figure, panel B). His condition had been treated with various antimicrobial agents without any result. Blood testing showed minor anemia (hemoglobin 12.4 g/dL) and that the patient was HIV negative. Bacteriologic analysis found no gram-positive and gram-negative bacteria, and ZN staining showed the presence of acid-fast bacilli. He received the specific recommended treatment for *M. ulcerans* infection (antimicrobial drug regimen: rifampin, 10 mg/kg, and streptomycin, 15 mg/kg), and the lesions had receded 2 months later (Figure, panel C).

The identification of *M. ulcerans* was confirmed by PCR on the basis of the IS*2404* repeated insertion sequences of *M. ulcerans* as described by Stinear et al. (*8*). The positive results were confirmed by quantitative real-time PCR, in the Laboratory of Bacteriology at Central Hospitalier Universistaire, Angers, France, on 2 specific sequences: IS*2404* sequence and ketoreductase B domain of the mycolactone polyketide synthase gene from the plasmid pMUM001 (*9*).

According to WHO criteria, 2 confirmative test results should be obtained of 4 laboratory tests (ZN staining, positive culture of *M.ulcerans*, specific gene amplification, pathognomonic histopathologic features) to establish a definitive diagnosis (*7*). Concerning the 2 patients in this study, results of ZN staining and PCR were positive, thus confirming the diagnosis of Buruli ulcer. Samples were inoculated on Löwenstein-Jensen (LJ) media and incubated at 30°C for 2 months, but the culture did not grow the organism. This result could be accounted for by the paucity of bacilli in the samples. In conclusion, our study confirms that, although infrequently diagnosed, Buruli ulcer is an endemic disease in CAR.

Identification and control of Buruli ulcer remain difficult in CAR, where this disease is often not considered. Even with evocative clinical signs, confirmation of diagnosis by biological analysis is still not easy. It is therefore of high importance that the public health authorities are fully informed and properly trained to identify this neglected disease in the early stages so patients can be cured before the onset of functional impairment and the appearance of extensive lesions. Further investigation to isolate strains present in CAR is also essential.
